# Otitis media por *Vibrio cholerae* no-O1/O139: Caso y revisión breve

**DOI:** 10.23938/ASSN.1148

**Published:** 2025-11-19

**Authors:** Diego Fernando Regalado Bermeo, Leticia Armendáriz López, Marta Adelantado Lacasa

**Affiliations:** 1 Servicio Navarro de Salud-Osasunbidea Hospital Universitario de Navarra Servicio de Otorrinolaringología Pamplona Navarra España; 2 Servicio Navarro de Salud-Osasunbidea Hospital Universitario de Navarra Servicio de Microbiología Pamplona Navarra España; 3 Servicio Navarro de Salud-Osasunbidea Hospital Reina Sofía Servicio de Microbiología Tudela Navarra España

**Keywords:** Infección por *Vibrio cholerae*, *Vibrio cholerae* no O1, Otitis Media, Infection, *Vibrio cholerae*, *Vibrio cholerae* non-O1, Otitis Media

## Abstract

*Vibrio cholerae e*s un bacilo gramnegativo que se encuentra frecuentemente en ambientes marinos. Los serotipos O1 y O139 están asociados con enfermedad entérica grave. Los serotipos no-O1/no-O139 son responsables de gastroenteritis leve pero también de bacteriemia e infecciones en heridas, oído y otras localizaciones.

Presentamos un caso de otitis media por *Vibrio cholerae* no-O1/no-O139 sensible a ciprofloxacino (MIC ≤0,5 µg/mL) en una paciente sin antecedente de viaje a zonas endémicas, con buena evolución con tratamiento tópico. Este caso sin exposición acuática clara y alejado de zonas endémicas contribuye a la presunción de que la otitis crónica por *Vibrio cholerae* fuera de entornos endémicos está probablemente infradiagnosticada y en ascenso, indicando la necesidad de cultivos y tratamientos dirigidos por antibiograma en casos de otitis de evolución tórpida.

## Abstract (en)

*Vibrio cholerae* is a gram-negative rod commonly found in marine environments. Serogroups O1 and O139 are associated with serious enteric disease, whereas non-O1/non-O139 serogroups are responsible for mild gastroenteritis, as well as bacteraemia and infections of wounds, the ear, and other sites.

We report a rare case of otitis media caused by non-O1/non-O139 *Vibrio cholerae* in a female patient without any history of travel to endemic areas, who showed favourable progress with topical treatment. This case, occurring without clear aquatic exposition and outside endemic regions, supports the presumption that chronic otitis caused by *V. cholerae* may be underdiagnosed and increasing in frequency, It highlights the importance of obtaining cultures and guiding treatment based on antibiotic susceptibility in cases of slow-onset otitis.

## INTRODUCCIÓN

*Vibrio cholerae* es un bacilo Gram negativo, con forma de *coma*, móvil y anaerobio facultativo, habitual de aguas salobres y preferentemente cálidas, ampliamente diseminado por el mundo^1^^,^^2^^,^^4^^-^^15^. Es conocido por causar infecciones gastrointestinales con potencial epidémico, específicamente las cepas O1 y O139 causantes de cólera. Sin embargo, otras cepas conocidas como NOVC (*Vibrio cholerae* no-O1/O139) generan desde infecciones leves con tratamiento relativamente sencillo hasta infecciones graves potencialmente letales^1^^-^^15^. Se han descrito 206 serogrupos según su antígeno mayor de superficie, el antígeno O. Mientras los serogrupos O1 y O139 poseen toxina colérica y pili co-regulados por toxina, que les confieren potencial epidémico, las cepas NOVC, también llamadas no aglutinantes, no los poseen, a excepción de las cepas O141, O75, O37, O10, O12, O6, y O14 que poseen toxina colérica^1^^,^^2^^,^^4^^-^^8^.

La principal manifestación de la infección por NOVC es una infección gastrointestinal que cursa con cuadro pseudocolérico generalmente autolimitado, más frecuente en meses de verano^10^^-^^15^, sin potencial epidémico. Sin embargo, también se reportan infecciones óticas, tanto medias como externas y, en algunos casos graves se ha reportado fascitis necrotizante y sepsis^1^^,^^2^^,^^7^^-^^15^. Hay que destacar que cada vez son más frecuentes los contagios en zonas no relacionadas con entornos de agua salada o endémicas de cólera^3^^,^^14^.

La descripción de este caso aporta información valiosa para el estudio y tratamiento dirigido por resultados microbiológicos de las infecciones óticas por NOVC, reforzando la pertinencia de considerarlas en el diagnóstico diferencial de otitis media crónica refractaria.

## CASO CLÍNICO

Presentamos el caso de una paciente de 36 años, natural de Marruecos, residente en la Ribera de Navarra, que en septiembre fue referida desde atención primaria al servicio de otorrinolaringología del hospital Reina Sofía de Tudela debido a la presencia de tapones de cera bilaterales y patología otológica crónica, para limpieza y seguimiento.

La paciente refirió otitis media crónica desde la infancia en seguimiento por otorrinolaringología en su país de origen, con episodios intermitentes de activación (otalgia y otorrea) durante años. En la anamnesis destaca ausencia de viaje a su país natal o fuera de España, hasta tres años antes de la consulta. El único contacto destacado con fuentes de agua corresponde a baño en aguas termales de la localidad, en el periodo de verano, no hay otros antecedentes relevantes más allá de hipotiroidismo de años de evolución para el cual se administra levotiroxina. 

En la exploración física, una vez limpios los conductos auditivos externos, se apreciaba perforación timpánica subtotal derecha húmeda, oído contralateral normal. Dada la cronicidad del caso, se decidió muestrear la otorrea por hisopado, aspirando los restos remanentes en la caja timpánica y conducto externo. Se inició tratamiento empírico con ciprofloxacina más corticoesteroide tópico (gotas) cada 12 horas y durante 7 días.

En el cultivo de la secreción se aisló *V. cholerae*. La identificación inicial se realizó con MicroScan WalkAway® 96 Plus (panel Negative NF Combo 71) y se confirmó en el Centro Nacional de Microbiología (Instituto de Salud Carlos III) como *V. cholerae* no-O1/no-O139 (gen ctx negativo).

Su sensibilidad a antibióticos se interpretó bajo criterios del *European Committee on Antimicrobial Susceptibility Testing* (EUCAST 2020, versión 10.0) y del *Clinical Laboratory Standards Institute* (CLSI M45); las concentraciones mínimas inhibitorias frente a las que la bacteria mostró sensibilidad fueron: <=8µg/mL para Amikacina, Ampicilina/sulbactam, y Piperacilina/Tazobactam; <=2 µg/mL para Trimethoprim/Sulphamethoxazole; <=1µg/mL para Cefepime, Ceftazidim, Imipenem, Levofloxacino, Meropenem y Tigeciclina; y <=0,5µg/mL para Ciprofloxacino.

Como parte del seguimiento del caso, y a partir de los resultados microbiológicos, se decidió mantener el tratamiento tópico y, 20 días después del inicio del mismo, realizar nuevo hisopado del conducto auditivo externo; en el cultivo únicamente se identificó flora habitual local.

Una vez resuelta la otorrea y la otalgia, se realizó audiometría tonal, donde se constató leve hipoacusia de transmisión relacionada con la perforación timpánica, quedando pendiente de reconstruir el defecto anatómico.

## DISCUSIÓN

Las infecciones óticas causadas por NOVC^2^^,^^3^^,^^7^^,^^10^ son una rara entidad clínica probablemente infradiagnosticada debido a la falta de realización de cultivos de rutina en casos de otitis media no complicada.

Aunque el *Vibrio cholerae* es un patógeno ampliamente conocido del cual se conocen muy bien sus métodos de aislamiento, cultivo y diferenciación genética en el contexto del cólera, cada vez es más común la comunicación de enfermedades originadas por *V. cholerae* no relacionadas con los serotipos O1 y O139. En la bibliografía analizada se documenta la creciente aparición de cepas NOVC en relación con el cambio climático^14^, siendo este un potencial campo de investigación ya que la capacidad del germen de mutar por transferencia horizontal de genes o recombinación genética^6^^,^^7^^,^^11^ podría, en un futuro, dar origen a cepas multiresistentes^10^^,^^11^^,^^13^.

La infección por cepas NOVC se asocia con factores de riesgo como edad mayor de 60 años^1^, inmunosupresión^14^^-^^15^, diabetes^8^^,^^14^^,^^15^, tumores hematológicos^8^ y enfermedad hepática (cirrosis)^14^^-^^15^; pudiendo multiplicarse por 80 el riesgo de contraer infecciones severas por NOVC en estos pacientes^1^^-^^3^.

**Tabla 1:** Relación de casos publicados de otitis causada por *Vibrio cholerae* no-O1/O139, incluyendo el presente caso

Fecha	Edad (años)	Tipo de otitis	Posible lugar de contagio	Referencia
	Sexo			
-	10	Media Crónica	Archipiélago Báltico (Suecia)	Back y col (1974)
	Masculino			
-	-	Media	-	Hughes y col (1978)
-	12	Externa Crónica	-	Hansen y col (1979)
	Femenino			
-	-	Media	-	Florescu y col (1981)
-	20	Media	Piscina (Bélgica)	Thibaut y col (1986)
	Femenino			
-	15	Externa	-	
	Masculino			
-	-	Media	-	Handrick y col (2004)
13/12/2000	3	Media Crónica	-	Huhulescu y col^7^^)^ (2007)
	Femenino			
29/12/2000	14	Externa	Lago Neusiedl (Austria)	
	Masculino			
11/05/2001	49	Media	-	
	Masculino			
31/07/2001	22	Externa	-	
	Masculino			
27/08/2004	9	Media	Lago Neusiedl (Austria)	
	Masculino			
13/09/2005	9	Media	Lago Neusiedl (Austria)	
	Femenino			
-	-	Otitis	-	Marek y col (2013)
2015	21	Externa	Lago Neusiedl (Austria)	Hirk y col (2016)
	Femenino			
2011	27	Media	Rio Murray (Australia)	Kechker y col (2017)
	Masculino			
2017	35	Externa	Mar Mediterráneo (España)	Díaz-Menéndez y col (2018)
	Masculino			
2017	11	Media	Lago Apremont (Francia)	Leroy y col (2019)
	Masculino			
2019	5	Media Crónica	Mar Báltico (Alemania)	Van Bonn y col (2020)
	Femenino			
1995	-	Otitis	Mar Báltico (Alemania)	Zhang y col^11^ (2024)
1995	-			
2012	-			
2017	-			
2021	36	Media Crónica	Arnedillo (La Rioja)	Caso actual
	Femenino			

Se realizó una búsqueda bibliográfica de los casos publicados en PubMed (“*otitis caused by Vibrio cholerae*”). En 2017, Kechker y col^5^ publicaron una serie de casos que incluía tanto casos históricos como la serie de Huhulescu y col^7^^)^ publicada en 2007. La Tabla 1 recoge estos casos y los publicados con posterioridad (con excepción de uno cuya finalidad radicaba en estudios genéticos), y añade el presente caso. Los resultados muestran un amplio rango de edad (5-49 años), con ligero predominio del sexo masculino (56,3%) y de personas ≤20 años (62,5%); un tercio de las descripciones no incluían sexo ni edad. Hubo exposición al agua en todos los casos en los que constaba el posible lugar de contagio. Más de la mitad de las infecciones eran otitis media (54,2%) y el resto otitis externa (25%) y casos en los que no se especificó la localización (20,9%).

De manera general, y salvo en casos de cólera graves, el uso de antibióticos no está indicado en el tratamiento de la enfermedad gastrointestinal; sin embargo, este no es el caso cuando las infecciones son extraintestinales^6^. El tratamiento de la infección ótica por organismos NOVC no representa conflictos, dada la sensibilidad a los antibióticos de uso estándar en otorrinolaringología. En la mayoría de casos descritos se empleó únicamente ciprofloxacino tópico^3^^-^^5^, igual que en nuestro caso, mientras que en otros se combinó con una cefalosporina sistémica de cara a la reconstrucción del defecto de caja y tímpano^4^, procedimiento que, realizado una vez confirmada la erradicación del germen causal, no presenta complicaciones ni en niños ni en adultos^2^^,^^4^^,^^5^.

Las descripciones de resistencias de cepas de *V. cholerae* hacen hincapié respecto a ampicilina, cloranfenicol, trimetoprim-sulfametoxazol y doxiciclina, encontradas en cepas de reservorios ambientales claramente vinculadas con el uso de antibióticos en la producción industrial de alimentos^6^. Otros estudios han descrito disminución de sensibilidad a gentamicina, azitromicina, ciprofloxacina, cefotaxima y eritromicina; sin embargo, los criterios usados por estos autores para la interpretación de resultados no son específicos para *V. cholerae*^7^^,^^10^.

En conclusión, la presentación de este caso sin exposición marina clara y alejado de zonas endémicas apoya la presunción de que la otitis crónica por *Vibrio cholerae* está probablemente subestimada. También remarca la necesidad de realizar hisopados rutinarios en cuadros de otitis externas recurrentes u otitis medias crónicas de evolución tórpida. La evolución favorable con tratamiento tópico apoya considerar las quinolonas como opción terapéutica, siempre respaldada por estudio microbiológico.

## Data Availability

Los datos clínicos, obtenidos del Sistema de Historia clínica Informatizada del Servicio Navarro de Salud-Osasunbidea, no están disponibles.

## References

[1] Vezzulli L, Baker-Austin C, Kirschner A, Pruzzo C, Martinez-Urtaza J (2020). Global emergence of environmental non-O1/O139 Vibrio cholerae infections linked with climate change: A neglected research field?. Environ Microbiol.

[2] Leroy AG, Lerailler F, Quilici ML, Bourdon S (2019). Vibrio cholerae non-O1/non-O139 otitis in metropolitan France. Med Mal Infect.

[3] Díaz-Menéndez M, Alguacil-Guillén M, Bloise I, García-Pallarés M, Mingorance J (2018). A case of otitis externa caused by non-01/non-0139 Vibrio cholerae after exposure at a mediterranean bathing site. Rev Esp Quimioter.

[4] Van Bonn SM, Schraven SP, Schuldt T, Heimesaat MM, Mlynski R, Warnke PC (2020). Chronic otitis media following infection by non-O1/non-O139 Vibrio cholerae: A case report and review of the literature. Eur J Microbiol Immunol (Bp).

[5] Kechker P, Senderovich Y, Ken-Dror S, Laviad-Shitrit S, Arakawa E, Halpern M (2017). Otitis media caused by V. cholerae O100: A case report and review of the literature. Front Microbiol.

[6] Laviad-Shitrit S, Sharaby Y, Izhaki I, Peretz A, Halpern M (2018). Antimicrobial susceptibility of environmental Non-O1/non-O139 Vibrio cholerae isolates. Front Microbiol.

[7] Huhulescu S, Indra A, Feierl G, Stoeger A, Ruppitsch W, Sarkar B (2007). Occurrence of Vibrio cholerae serogroups other than 01 and 0139 in Austria. Wien Klin Wochenschr.

[8] Maraki S, Christidou A, Anastasaki M, Scoulica E (2016). Non-01, non-0139 Vibrio cholerae bacteremic skin and soft tissue infections. Infect Dis (Lond).

[9] Rodríguez JY, Duarte C, Rodríguez GJ, Montaño LA, Benítez-Peñuela MA, Díaz P (2023). Bacteremia by non-O1/non-O139 Vibrio cholerae: Case description and literature review. Biomedica.

[10] Wu Q, Vaziri AZ, Omidi N, Hassan Kaviar V, Maleki A, Khadivar P (2023). Antimicrobial resistance among clinical Vibrio cholerae non-O1/non-O139 isolates: Systematic review and meta-analysis. Pathog Glob Health.

[11] Zhang Q, Alter T, Strauch E, Eichhorn I, Borowiak M, Deneke C (2024). German coasts harbor non-O1/non-O139 Vibrio cholerae with clinical virulence gene profiles. Infect Genet Evol.

[12] Brehm TT, Berneking L, Sena Martins M, Dupke S, Jacob D, Drechsel O (2021). German Vibrio study group. Heatwave-associated Vibrio infections in Germany, 2018 and 2019. Euro Surveill.

[13] Deshayes S, Daurel C, Cattoir V, Parienti JJ, Quilici ML, de La Blanchardière A (2015). Non-O1, non-O139 Vibrio cholerae bacteraemia: Case report and literature review. Springerplus.

[14] Laurens S, Wilhelm N, Février F, Coppin D, Mothes M, Quilici ML (2024). Non-O1/non-O139 Vibrio cholerae bacteremia and hepatitis in diabetic patient, France, winter 2019. Diagn Microbiol Infect Dis.

[15] Chen YT, Tang HJ, Chao CM, Lai CC (2015). Clinical manifestations of non-O1 Vibrio cholerae infections. PLoS One.

